# The Circadian Deadenylase Nocturnin Is Necessary for Stabilization of the *iNOS* mRNA in Mice

**DOI:** 10.1371/journal.pone.0026954

**Published:** 2011-11-02

**Authors:** Shuang Niu, Danielle L. Shingle, Eduardo Garbarino-Pico, Shihoko Kojima, Misty Gilbert, Carla B. Green

**Affiliations:** 1 Department of Biology, University of Virginia, Charlottesville, Virginia, United States of America; 2 Department of Neuroscience, University of Texas Southwestern Medical Center, Dallas, Texas, United States of America; Vanderbilt University, United States of America

## Abstract

Nocturnin is a member of the CCR4 deadenylase family, and its expression is under circadian control with peak levels at night. Because it can remove poly(A) tails from mRNAs, it is presumed to play a role in post-transcriptional control of circadian gene expression, but its target mRNAs are not known. Here we demonstrate that *Nocturnin* expression is acutely induced by the endotoxin lipopolysaccharide (LPS). Mouse embryo fibroblasts (MEFs) lacking *Nocturnin* exhibit normal patterns of acute induction of *TNFα* and *iNOS* mRNAs during the first three hours following LPS treatment, but by 24 hours, while *TNFα* mRNA levels are indistinguishable from WT cells, *iNOS* message is significantly reduced 20-fold. Accordingly, analysis of the stability of the mRNAs showed that loss of *Nocturnin* causes a significant decrease in the half-life of the *iNOS* mRNA (t_1/2_ = 3.3 hours in Nocturnin knockout MEFs vs. 12.4 hours in wild type MEFs), while having no effect on the *TNFα* message. Furthermore, mice lacking *Nocturnin* lose the normal nighttime peak of hepatic *iNOS* mRNA, and have improved survival following LPS injection. These data suggest that Nocturnin has a novel stabilizing activity that plays an important role in the circadian response to inflammatory signals.

## Introduction

Living organisms require dynamic changes in protein composition to correspond to changes in the environment. Some of these changes are unpredictable – such as exposure to pathogens or toxins, while others are predictable – such as the daily cyclic changes in light levels and nutrient availability. Many types of acute signaling systems are in place to mediate the biological responses to the former, while the circadian system regulates the response to the latter, driving daily temporal changes in protein type and quantity to allow the organism to coordinate physiological events with time of day. In addition, interactions between the systems also occur since some acute responses can alter the circadian system, and the circadian system can cause changes in the magnitude and types of acute responses at different times of day. Although transcriptional changes are important for both types of response systems, it has become increasingly clear that post-transcriptional mechanisms play a critical role in dictating the protein complement of the cell. Indeed, tight control of RNA stability and/or translatability provides a way to rapidly modulate responses and quickly terminate protein production.

Nocturnin is a deadenylase, a 3′ exoribonuclease with specificity for tracts of poly(A), that is under both circadian and acute regulation. Identified originally as a rhythmic mRNA in *Xenopus* retina [1], [2], [3], it is expressed with high amplitude rhythms in many tissues in mice with peak levels at night [4], [5]. In addition to this circadian expression, it is also acutely inducible by a number of stimuli, exhibiting immediate early gene responses [6]. Mice lacking this gene have a metabolic phenotype, resistance to diet-induced obesity, and changes in rhythmic expression of metabolic-related genes in several different tissues/cell types [7]. Therefore, based on Nocturnin's known activity and expression pattern, our working hypothesis is that Nocturnin is involved in the post-transcriptional regulation of both rhythmic and acutely inducible mRNAs. However the specific target mRNAs upon which Nocturnin acts are not known.

Deadenylation, or poly(A) tail removal, is a rate-limiting and highly regulated step in the mRNA decay pathway, and also can regulate translatability of the message [8], [9], [10]. Nocturnin is a member of the CCR4 family of deadenylases, of which there are 5 in mice and humans [11], [12]. A single Ccr4p protein exists in yeast and interacts with Caf1p/Pop2p proteins as part of the Ccr4-Not complex – the major deadenylase in yeast [13], [14], [15]. In mammals, the members of this family all contain a conserved Mg^++^-dependent nuclease domain similar to that found in yeast Ccr4p and typified by the apurinic/apyrimidinic endonuclease (APE) family of proteins [11], but only two of the members of this family (called “Ccr4a” and “Ccr4b” in humans) have N-terminal extensions that contain leucine-rich repeats like those necessary for the Caf1p/Pop2p interaction in yeast. The other three members, including Nocturnin (also known as “hCcr4c” in humans) are shorter, lacking these N-terminal domains. One of these short Ccr4s in humans (hCcr4d) has been shown to be part of an “unconventional” deadenylase complex found in nuclear Cajal bodies [12] and it has been proposed by these authors that the other short Ccr4s may also have diverged to take on specialized functions. Although recombinant mouse Nocturnin has been shown to possess *bona fide* deadenylase activity [6], the *in vitro* activity is not robust compared to other deadenylases, and the human Nocturnin has been reported to lack activity, at least within the conditions of the *in vitro* assays and using generic substrates [12].

Because Nocturnin is an immediate early gene, and its expression is up-regulated by many acute stimuli [4], [6], we examined the promoter region of the *Nocturnin* gene to identify the *in vivo* signals responsible for *Nocturnin's acute* induction. This led us to the discovery of multiple nuclear factor-kappa B (NF-κB) sites, indicating Nocturnin might be induced in response to inflammatory signals. Since many mRNAs involved in the inflammatory response are highly regulated and under post-transcriptional control, we used a candidate approach to explore whether Nocturnin expression altered the stabilization of these transcripts. This led to the surprising discovery that the presence of Nocturnin stabilizes the pro-inflammatory transcript, *iNOS*, rather than destabilizing it. This results in significant *in vivo* changes in *iNOS* expression profiles that may impact how the circadian clock modulates the inflammatory response of the animal.

## Results

### Nocturnin is induced by LPS

**Figure 1:**
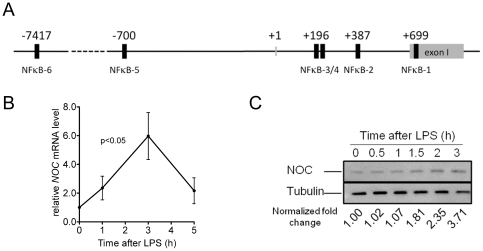
*Nocturnin* is induced by LPS in MEFs. (**A**) Putative NF-κB enhancer sites located in conserved regions in the *Nocturnin* gene and flanking sequence. Human and mouse genomic sequences spanning 10 Kb upstream of the *Nocturnin* gene were aligned and analyzed by Matlnspector [48]. Shown are relative positions of predicted conserved NF-κB binding sites (black boxes) near the mouse *Nocturnin* gene. +1 denotes transcription start site and negative and positive numbers denote nucleotide position within the mouse sequence relative to that site. (**B,C**) MEFs treated with LPS for indicated times were collected for total RNA isolation and protein extraction. *iNOS* mRNA levels were determined by quantitative RT-PCR and normalized to *Gapdh* mRNA level. Data represent mean (±SEM) of three independent MEF lines. (B). NOC protein levels were determined by Western blotting and normalized to Tubulin. The fold changes of NOC protein at different time points are indicated (C).

Nocturnin has been shown to be rapidly induced in response to various acute signals in culture, but *in vivo* signals that induce Nocturnin are not well characterized [6]. We analyzed the genomic regions 10 kb upstream from the murine and human *Nocturnin* translation start sites to identify putative enhancer sites that may regulate *Nocturnin'*s acute induction. This analysis revealed many sequences with high similarity to canonical NF-κB transcription binding sites (Figure 1A). Since NF-κB is a central regulator of inflammatory responses, we investigated whether lipopolysaccharide (LPS), could induce *Nocturnin* expression. Indeed, we observed a 5–6 fold induction of the *Nocturnin* mRNA and a 3-fold increase in Nocturnin protein levels in mouse embryonic fibroblasts (MEFs) within 3 hours following LPS treatment (Figure 1B–C).

### Nocturnin is necessary for the normal accumulation of iNOS protein following LPS stimulation

**Figure 2:**
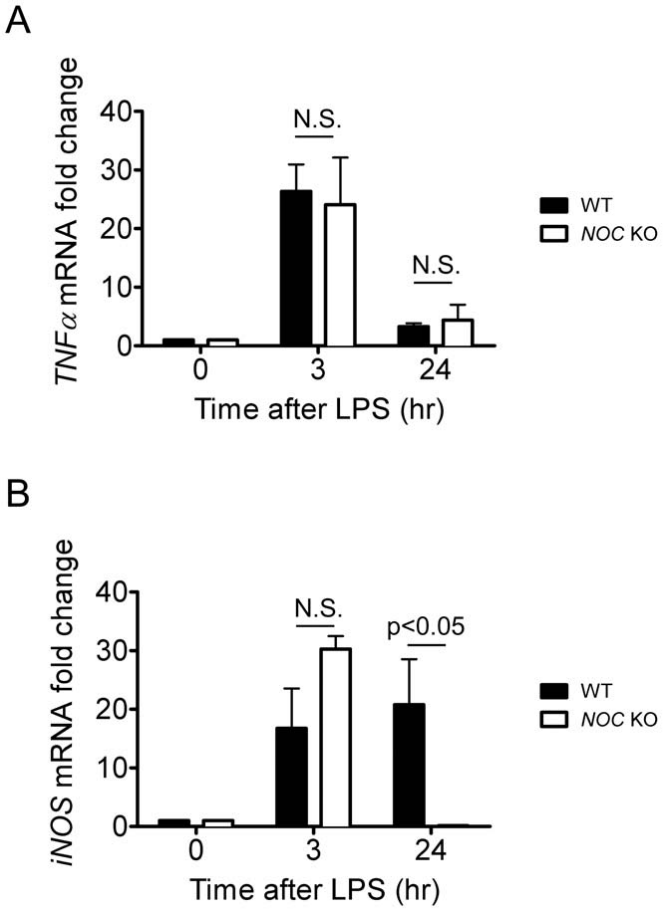
*iNOS* mRNA is induced by LPS, but does not accumulate in *Noc* KO MEFs. MEFs treated with LPS for the indicated times were collected for total RNA isolation. mRNA levels of *TNFα* (**A**) and *iNOS* (**B**) were determined by quantitative RT-PCR and normalized against *β2 microglobin*. Data represent mean (±SEM) of three independent MEF lines and comparison between genotypes at each time point was done by two-way ANOVA followed by Bonferroni post tests (Prism). N.S. = no significant difference between genotypes (p>0.05).

**Figure 3:**
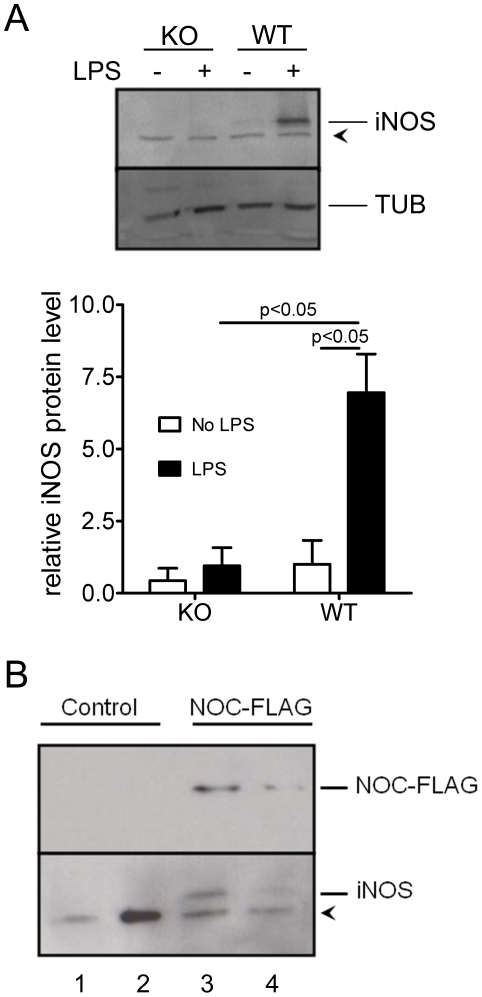
Accumulation of *iNOS* protein following LPS requires Nocturnin. (**A**) iNOS protein is reduced in *Noc* KO MEFs following LPS treatment. WT and *Noc* KO MEFs treated with LPS for twenty-four hours were collected for protein extraction. Proteins were analyzed by Western blotting with α-iNOS antibody (a representative blot is shown in top panel) and protein levels were determined (normalized to β-tubulin levels) and plotted as mean (±SEM) of three independent MEF lines (bottom panel). (**B**) Expression of Nocturnin in *Noc* KO MEFs rescues the LPS induction of iNOS protein. *Noc* KO MEFs were electroporated with either empty FLAG vector (control) or a vector expressing a FLAG-Nocturnin fusion protein (NOC-FLAG). Forty-eight hours after electroporation, MEFs were treated for 24 hours with LPS and were then collected for protein extraction. Proteins were analyzed by Western blotting with α-iNOS and α-FLAG antibodies. Shown are extracts expressing two different levels of NOC-FLAG (compare lanes 3 and 4, top blot), which correlate with the level of rescued iNOS protein (compare lanes 3 and 4, bottom blot). The arrowhead marks a non-specific band (this band is not iNOS, as it is still present in samples from iNOS knockout mice, data not shown). The data shown are from two independently-derived WT and two independently-derived KO MEF lines (one per lane). This experiment was also repeated in a third independent MEF line for each genotype with similar results (data not shown).

The observation that Nocturnin is induced by LPS led us to investigate whether Nocturnin plays a role in regulating the LPS-induced inflammatory response. To do this, we examined the induction profiles of *TNFα and iNOS*, two known LPS-responsive mRNAs, in wild-type (WT) and *Noc* KO MEFs. Both of the mRNAs showed significant induction by LPS after 3 hours in both the WT and *Noc* KO MEFs, and this induction was not significantly different between genotypes (Figure 2). By 24 hours after LPS treatment, the mRNA levels of *TNFα* decreased in both WT and *Noc* KO cells, returning nearly to basal levels (Figure 2A). In contrast, although the *iNOS* mRNA continues to rise in the WT cells over 24 hours, it was nearly undetectable in the *Noc* KO cells (Figure 2B). The *Noc* KO cells also had undetectable levels of the iNOS protein 24 hours following LPS, in contrast to the high levels observed in the WT cells at that time point (Figure 3A). To verify that the loss of iNOS induction in the KO MEFs was due solely to the lack of Nocturnin, we expressed heterologous Nocturnin protein in the *Noc* KO cells. Nocturnin expression was sufficient to rescue iNOS protein induction by LPS, and did so in a dose-dependent manner (Figure 3B). This indicates that Nocturnin is necessary for the normal accumulation of iNOS protein following LPS stimulation in MEFs.

### Nocturnin stabilizes iNOS mRNA

**Figure 4:**
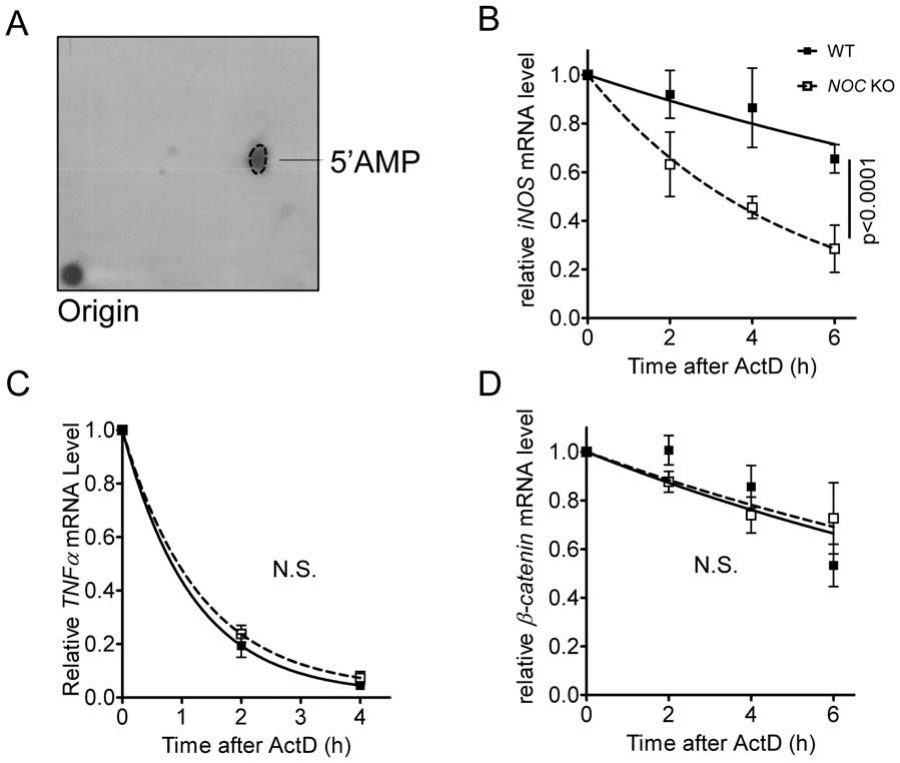
Nocturnin alters *iNOS* mRNA half-life. (**A**) Nocturnin-mediated deadenylation releases 5′AMP. Recombinant GST-NOC protein was incubated with an RNA substrate containing a 100 nt poly(A) tail labeled with [α-^32^P] -ATP at 37°C for 30 min. The radiolableled product of the reaction was determined by two-dimensional thin layer chromatography analysis as described in the Materials and Methods. The dotted line denotes the position of the cold 5′AMP marker. (**B–D**) The stability of the *iNOS* mRNA (**B**), but not *TNFα* (**C**) or *β-catenin* (**D**) is significantly reduced in the MEFs lacking Nocturnin. MEFs were treated with LPS for 3 hours before the experiment. At time 0, LPS was removed and replaced with media containing actinomycin D and cells were collected for RNA isolation at the times indicated. mRNA levels were determined by quantitative RT-PCR and normalized against *Gapdh* mRNA. In each case, the mRNA level at time 0 was set as 100% and data were plotted as mean (±SEM) of 4–6 independent MEF cell lines for each genotype.

Nocturnin has deadenylase activity in *in vitro* experiments, removing 5′adenosine monophosphates from the 3′ end of polyadenylated substrates [Figure 4A; [1], [6]]. Removal of the poly (A) tail from transcripts typically alters the stability of the mRNAs, causing them to degrade more rapidly. To determine whether Nocturnin influences the *iNOS* mRNA half-life, we treated WT and *Noc* KO MEFs with LPS for 3 hours to induce *iNOS* mRNA and then blocked transcription with the addition of Actinomycin D and collected samples at different intervals to measure mRNA stability. In these experiments, we saw a clear and surprising effect of Nocturnin on the half-life of *iNOS* mRNA (Figure 4B). The *iNOS* mRNA was significantly less stable (t_1/2_ = 3.3 hours vs. 12.4 hours) in the MEFs lacking Nocturnin, suggesting that the presence of Nocturnin has a stabilizing effect on *iNOS* message. In the *Noc* KO MEFs we did not observe any change in the half-lives of *TNFα* mRNA (Figure 4C) or *β-catenin* (Figure 4D), a message unrelated to the inflammatory response, indicating that the Nocturnin-dependent stabilization of *iNOS* is transcript specific and is not a general effect.

### Nocturnin does not localize in mRNA-storage cytoplasmic subdomains or polysomes

**Figure 5:**
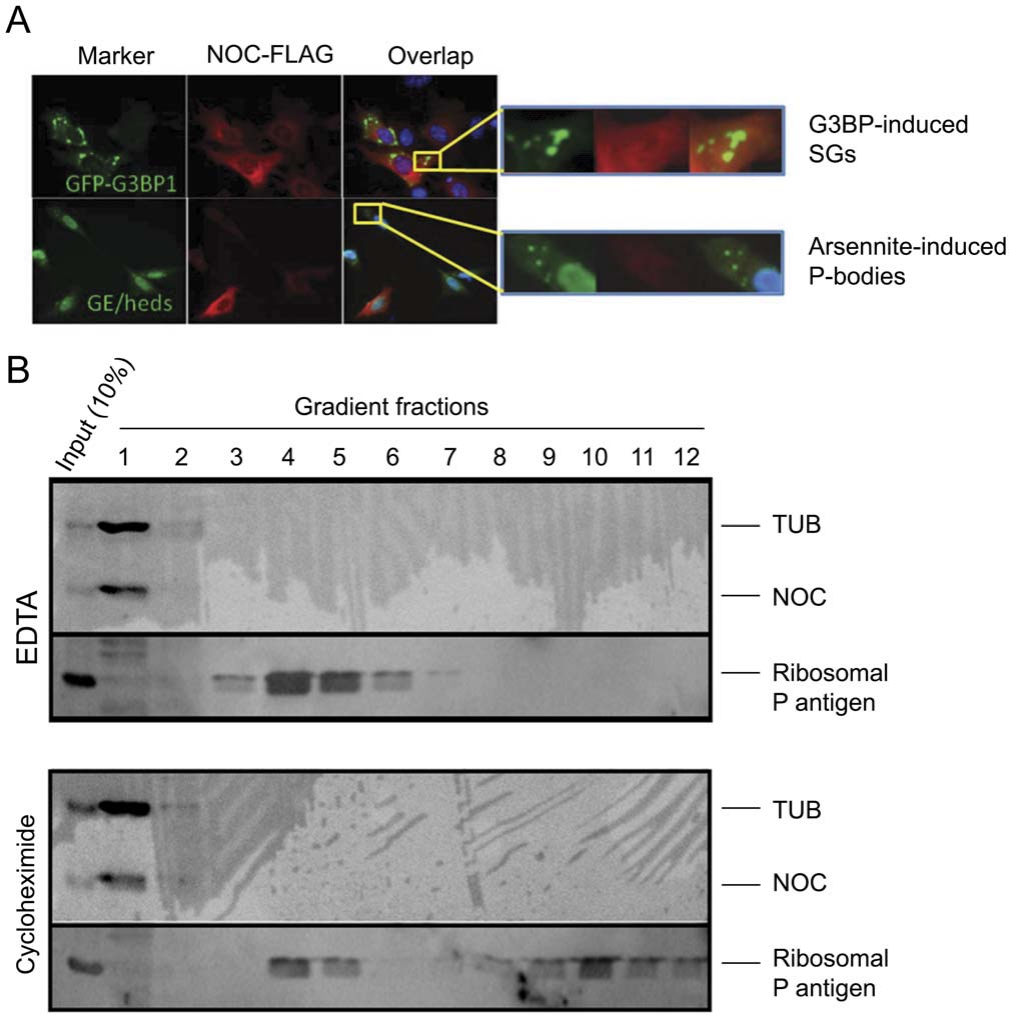
Subcellular localization of Nocturnin protein. (A) Nocturnin protein does not co-localize with stress granules (SGs) or P-bodies (PBs). NIH3T3 cells were transfected with NOC-FLAG only (arsenite-induced SG and PB experiments) or pE-eGFP-G3BP1 and NOC-FLAG (G3BP1-induced SG experiments) constructs. Specific antibodies were used to detect NOC-FLAG and GE-heds (marker for P-bodies). Part of the image was amplified on the right to more clearly show the lack of overlap. Shown are representative examples from 3 independent experiments. (B) Nocturnin protein does not co-localize with polysomes. Polysome analysis was conducted by sucrose gradients. EDTA or cycloheximide was utilized to disassociate or freeze the polysomes, respectively. Fractions were collected and separated by SDS-PAGE and analyzed by Western blotting. Tubulin and ribosomal P antigen were blotted as markers of non-polysome fractions and ribosome/polysome fractions, respectively. This fractionation was performed on two independent occasions with indistinguishable results.

Considering that the deadenylase CCR4a/b can localize to processing bodies (P-bodies; [16], [17]), that a number of translationally silenced mRNAs can be stored in a deanylated form in P-bodies or stress granules —two cytoplasmic subdomains that are important for RNA storage, decay, and translational silencing [18], [19], and that the stabilized *iNOS* mRNA may be stored in those foci, we analyzed whether Nocturnin localizes to some of these structures. However, Nocturnin does not colocalize with markers of stress granules or P-bodies (Figure 5A). Additionally, we hypothesized that Nocturnin may deadenylate the *iNOS* mRNA in polysomes, inducing changes in its translational status, but polysome analysis demonstrated that Nocturnin is not present in polysomes (Figure 5B). This implies that Nocturnin may be functioning outside of the known mRNA processing pathways.

### Nocturnin regulates iNOS rhythmicity

**Figure 6:**
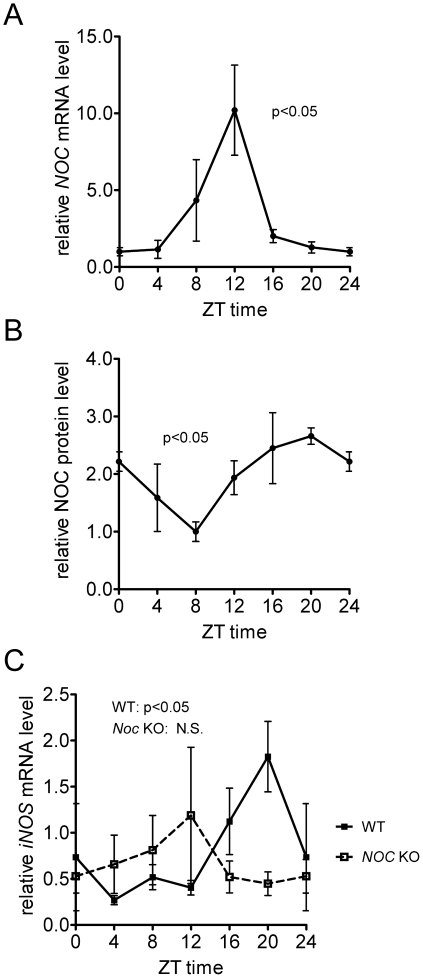
Nocturnin regulates rhythmic expression of *iNOS* in mouse liver. (**A,B**) *Nocturnin* mRNA and protein are rhythmic in mouse liver. Mice were maintained on a 12∶12 light-dark cycle, and liver samples were collected at different times of day as indicated. “ZT” refers to Zeitgeber Time (hrs), where ZT0 is defined as light onset and ZT12 is defined as dark onset. (**A**) *Nocturnin* mRNA levels were determined by quantitative RT PCR and normalized against *β-2 microglobin*. (**B**) Nocturnin protein levels were determined by measuring the chemiluminesence density from three independent western blots (n = 3 mice) and plotted as mean±SEM. (**C**) *iNOS* mRNA level is rhythmic in WT, but not in *Noc* KO, mouse livers. *iNOS* mRNA levels were measured in the same samples described in (A) by quantitative RT-PCR and normalized to *β-2 microglobin*. Data are presented as mean (±SEM) of 3–6 mice for each time point. For all the curves, data from ZT0 was re-plotted as ZT24 to improve visualization of the 24 hour cycle. Rhythmicity of curves was determined by CircWave v1.4 software (http:/hutlab.nl; R. A. Hut, University of Groningen).

We next examined whether Nocturnin controls *iNOS* levels *in vivo* by measuring the levels of *iNOS* mRNA in WT and *Noc* KO mouse livers, a tissue which expresses *iNOS* and in which Nocturnin shows a very high amplitude rhythm of expression [Figure 6A; [5]]. Analysis of the circadian profile of basal *iNOS* mRNA revealed high amplitude rhythms in the liver with peak levels occurring during the late night, when Nocturnin protein is highest (Figure 6B). In *Noc* KO mice, however, this rhythm is lost, demonstrating that Nocturnin protein is necessary for the nighttime accumulation of the *iNOS* mRNA (Figure 6C).

### Nocturnin is involved in the in vivo response to LPS challenge

**Figure 7:**
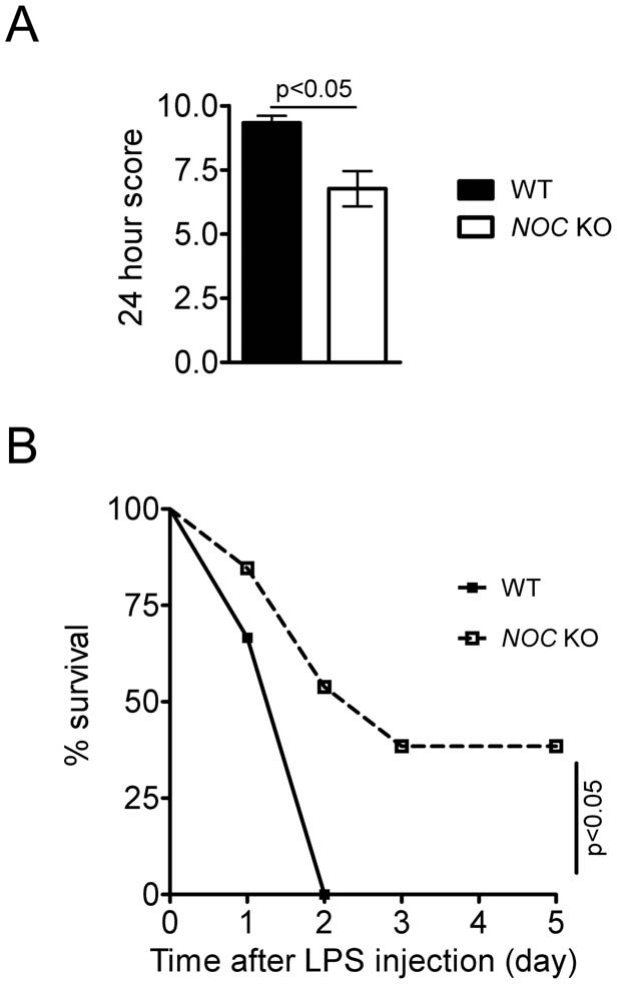
Noc KO mice are more resistant to an LPS challenge. (**A**) WT mice were more adversely affected than *Noc* KO mice 24 hours after LPS injection. Mice were injected with 10 mg/kg of body weight of LPS at ZT4, and “sickness” of mice was assessed using subjective scoring criteria (Materials and Methods). The score 24 hours after injection were plotted as mean (±SEM) of 6 (WT) and 12 (*Noc* KO) mice. (**B**) WT mice had a lower survival rate than *Noc* KO mice following LPS injection. Mice described in (A) were monitored for 5 days. All mice still alive on the fifth day post-injection had a sickness score less than 3 (recovered).

LPS-induced endotoxic shock, is widely used as a model of the inflammatory responses [20], [21], [22], [23], [24] and therefore, to further explore whether Nocturnin is involved in regulating an inflammatory response *in vivo*, we examined the survival rate of WT and Nocturnin KO mice following injection with LPS. Blind subjective scoring of “sickness,” 24 hours following the injection, demonstrated that the WT mice were significantly more adversely affected by the LPS injection than were the KO mice (Figure 7A). This genotype-specific difference continued for several days, with decreased “survival” in the WT mice (see Methods: Figure 7B).

## Discussion

We have shown that the stability of *iNOS* mRNA is regulated by the circadian deadenylase Nocturnin, and we have demonstrated that Nocturnin is necessary for the circadian rhythm of *iNOS* mRNA levels in the liver. Our data suggest that the *iNOS* rhythmicity is a result of a stabilization of the *iNOS* mRNA by Nocturnin during the night when Nocturnin levels are high. Regulation of iNOS at the level of mRNA stability has been described in many contexts including hepatocytes and Kupffer cells of the liver [25], [26]. The 3′UTR of the *iNOS* transcript contains several AU-rich elements (AREs) and several ARE-binding proteins have been shown to bind to these elements, including HuR, AU-binding factor 1/heterogeneous nuclear ribonucleoprotein D (hnRNP D), and KSRP [27], [28], [29]. Additionally, several other RNA binding proteins such as hnRNP I/polypyrimidine tract-binding protein (PTB) and hnRNP L also have been shown to interact with other regions of the *iNOS* 3′UTR and contribute to its proper post-transcriptional control [27], [30], [31]. In hepatocytes, *iNOS* mRNA levels are also regulated by a fascinating mechanism in which a natural antisense transcript of the *iNOS* gene interacts with the *iNOS* message, forming a direct binding platform for the HuR protein that stabilizes the message [32].

Why does the loss of Nocturnin shorten the half-life of the *iNOS* mRNA? Deadenylation is generally thought to be an important regulatory step for targeting a message for degradation [8], [9], and yet loss of the Nocturnin deadenylase causes the *iNOS* mRNA to degrade more rapidly, calling into question Nocturnin's *in vivo* role. However, our data do not discriminate between direct and indirect effects of Nocturnin on the *iNOS* message, and it is possible that Nocturnin is destabilizing some other message that encodes an *iNOS* destabilizer. However, the recent observation that many RNAs can exist in stable, but short-tailed forms [33] suggests that the role of deadenylation may be more complex than previously thought. Nevertheless, we have shown that Nocturnin does not localize to known RNA storage compartments such as stress granules or P-bodies, and is not present in polysomes. Also, loss of Nocturnin in MEFs results in loss of both *iNOS* mRNA and iNOS protein, suggesting that Nocturnin does not simply convert the *iNOS* message into a stable short-tailed but translationally silent form.

It is possible that Nocturnin competes with other more robust deadenylases for certain target mRNAs. In *in vitro* assays, Nocturnin has a less robust activity than other deadenylases such as PARN [6], although there are many caveats in interpreting such assays with generic substrates and isolated enzymes. Perhaps a “slow” or inefficient deadenylase protects messages from rapid deadenylation and subsequent decay. Repeated attempts to measure the poly(A) tail lengths of the *iNOS* mRNA in our *Noc* KO cells were unsuccessful due to the extremely low levels of *iNOS* message. But support for such an idea comes from analysis of the CCR4 family members in humans where the “yeast-like” members with the extended leucine-rich N-terminal domains (CCR4a and CCR4b) have robust deadenylase activity while the other members, including Nocturnin, do not. One of the members of this latter class, Ccr4d, was shown to participate in a deadenylation complex in nuclear Cajal bodies [12]. Perhaps Nocturnin has diverged to take on a role that stabilizes mRNAs, either through its deadenylase activity or some other unknown activity.

Recent reports have shown that the circadian clock modulates the innate immune system in physiologically important ways [34], [35]. Both the clock and the immune system are under significant post-transcriptional control [36], [37], [38], [39] although the inflammatory system has been much more extensively studied in this regard. Many mRNAs that encode inflammatory response proteins have short half-lives and this instability is necessary to avoid accumulation of potentially harmful protein products in times when they are not needed [39]. Our data strongly suggest that Nocturnin plays an important role in stabilization of the hepatic *iNOS* message both following LPS stimulation and during the night within the circadian cycle.

Why are the basal levels of *iNOS* mRNA rhythmic in the liver? Perhaps this is related to a circadian change in susceptibility to inflammatory signals. It has been reported that mice exhibit significantly higher lethality to LPS and TNFα administration during their resting phase (day) and that this rhythm is under circadian control [40], [41]. Our demonstration that mice lacking Nocturnin exhibit higher survivability following LPS injection suggests that Nocturnin is playing a role in this daily change in sensitivity. Whether this resistance to LPS is due to the changes we observe on *iNOS* stability is not yet known and further studies will be required to more carefully examine other aspects of the innate immune response in the *Noc* KO mice, and to determine whether other inflammatory signals may also be Nocturnin targets.

## Materials and Methods

### Plasmids

To generate the Noc-FLAG construct, annealed oligos encoding three copies of FLAG tag were inserted between XhoI and SalI sites of a pCMV-Tag4A vector (Stratagene). The mouse *Nocturnin* cDNA was then subcloned into the EcoRI and XhoI sites. Mouse *Noc* (GenBank Accession Number AF199491) was cloned in a pDEST15 vector (Invitrogen) for *Escherichia coli* expression with an N-terminal GST-tag. pE-eGFP-G3BP1 plasmid was a generous gift of Dr. Nancy Kedersha and Dr. Paul Anderson.

### Antibodies and Western blot

The custom α-Nocturnin antibody was raised in rabbits (using services provided by Sigma Life Sciences) against a recombinant mouse Nocturnin protein (NP_033964) starting from the amino acid 65 (methionine). The Ribosomal P antigen antibody was obtained from ImmunoVision, the α-FLAG antibody, the α-Tubulin antibody and the α-GE-heds antibody were obtained from Sigma and the α-iNOS antibody was purchased from Santa Cruz Biotechnology. All western blot signals were quantified by the Storm 820 Phosphorimager (GE Healthcare Life Sciences).

### Animals

WT and *Noc* KO (C57BL/6J background) mice were maintained under a 12∶12 h light-dark cycle with free access to food and water. For circadian profiles of protein and mRNA, liver tissue was collected at 4 hour intervals over 24 hours, dark time points were collected using infrared goggles.

### LPS treatment

Mice were injected intraperitoneally with 10 mg/kg body weight of LPS (Sigma) at ZT4. The “sickness” score and survival rate of mice were monitored for 5 days after LPS injection, and the experiment was terminated when all the animals alive had a score less than 3. The “sickness” scores were tallied with the observation time point (24 hours after LPS injection) according to the criteria listed below. All scoring was done “blind” with genotypes unknown to the scorer.

All LPS experiments were carried out under the supervision of veterinary staff,, and with the approval of the IACUC committee at University of Virginia. The number of animals in the procedure was kept to the smallest number necessary to obtain statistical confidence in the replicability of the results (WT 6 mice, KO 12 mice). We chose a commonly used dose (10 mg/kg body weight; [20], [21], [22]) for eliciting the endotoxic response. To minimize the pain and distress that animals might suffer, as well as for assessing the severity of the inflammatory response, a humane endpoint was determined by a small pilot study following the guideline of the IACUC committee in University of Virginia. The mice were monitored twice daily and softened food pellets were placed on the cage floor for easy access. A “sickness” score (see below) was given to each mouse during each monitoring session, and all scoring was done “blind” with genotypes unknown to the scorer. To prevent unnecessary suffering, any animal with a score of 8 or higher, and any mice with 2 or more maximum scores (in any category) were euthanized. These euthanized animals are included in the “dead” category in the survivability curve. The “sickness” score and survival rate of mice were monitored for 5 days after LPS injection, and the experiment was terminated when all the animals alive had a score less than 3. The “sickness” scores were tallied with the observation time point (24 hours after LPS injection) according to the criteria listed below.

*Sickness Scoring:*

*Body Weight Changes:* 0 = normal; 1 = <10% weight loss; 2 = 10–15% weight loss; 3 = >20% weight loss.
*Physical Condition, Haircoat:* 0 = normal, well-groomed; 1 = rough haircoat; 2 = rough coat, hair loss, ungroomed.
*Physical Condition, Eyes and Nose:* 0 = normal; 1 = eyes closed or squinted, no discharge; 2 = eyes closed or squinted, discharge or porphyrin staining
*Behavior, Activity:* 0 = normal; 1 = decreased activity, locomotion after slight stimulation; 2 = inactive, less alert, locomotion after moderate stimulation; 3 = self-mutilation, either very restless or immobile, or no locomotion after moderate stimulation.
*Behavior, Posture:* 0 = normal; 1 = sitting in hunched up position; 2 = hunched posture/head on cage floor; 3 = lying prone on cage floor.

### MEFs primary cell culture

Primary cultures of MEF cells from WT and *Noc* KO mice were made as described in [42] and maintained in MEF media (DMEM supplemented with 10% FBS, MEM nonessential amino acids, 2 mM L-glutamine, 0.1 mM 2-mercaptoethanol, 20 mM HEPES, pH7.3, and antibiotic-antimycotic mix solution). For LPS experiments, asynchronous MEFs were cultured in MEF media without FBS and phenol red for 18 hours before LPS treatment and then replaced with fresh media containing 5 µg/ml of LPS for indicated times. For mRNA decay experiments, MEF media (without FBS and phenol red) containing 1 µg/ml of actinomycin D was added upon the removal of the LPS media. For protein analysis, cells were lysed in T-PER buffer (Pierce) with protease inhibitor cocktail (Sigma) 24 hrs after LPS addition. Rescue experiments were conducted by electroporating *Noc* KO MEFs with NOC-FLAG or FLAG only expression vectors using a MEF1 nucleofector kit (Lonza). Forty-eight hours after electroporation MEFs were treated for 24 hours with LPS as described above and collected for protein extraction. Proteins were analyzed by western blotting with α-iNOS and α-FLAG antibodies.

### Quantitative reverse transcriptase PCR

Total RNA was isolated using Trizol reagent according to the manufacturer's protocol (Invitrogen) and reverse transcribed using random primers. The quantitative RT-PCR reaction was conducted with either SYBR Green (Invitrogen) or Taqman gene expression assay (ABI). The relative expression of indicated RNAs was normalized to an internal control using the Δ*Ct* method (*Gapdh* or *β2-microglobin)* or ΔΔCt as described by the ABI protocol respectively. Primers for *iNOS*, *β-Catenin*, *β2-microglobin*, and *Gapdh* are obtained from Harvard Primer Bank and the sequences are: *Noc* 5′-ACCAGCCAGACATACTGTGC-3′, 5′-CTTGGGGAAAAACGTGCCT-3′; *iNOS* 5′-GTTCTCAGCCCAACAATACAAGA-3′, 5′-GTGGACGGGTCGATGTCAC-3′; *β-Catenin* 5′-AGTTTCCTATGGGAACAGTCG-3′, 5′-AGGTTCACTAGAACATAACACT-3′; *β2-microglobin* 5′-TTCTGGTGCTTGTCTCACTGA-3′, 5′-CAGTATGTTCGGCTTCCCATTC-3′; *Gapdh* 5′-AGGTCGGTGTGAACGGATTTG-3′, 5′-TGTAGACCATGTAGTTGAGGTCA-3′. Taqman primer sets were purchased from Applied Biosystems: *TNFα*-Mm00443258_m1; *iNOS*- Mm00440485_m1; *β2-microglobin*- Mm00437762_m1.

### In *vitro* deadenylase assay and two-dimensional thin layer chromatography

The deadenylase assay was carried out as described previously [1], [6], [43], [44]. The RNA substrate was synthesized from the G52 plasmid containing the last 86 nt of the β-globin 3′UTR followed by a 100 nt poly(A) tail. Radiolabeled RNA substrate was synthesized by transcribing *BamHI* linearized DNA constructs with SP6 polymerase (GIBCO-BRL) in the presence of [α-^32^P]-ATP (800 Ci/mmol, Perkin Elmer) and 625 µM of a 5′ GpppG cap analog (Amersham Pharmacia Biotech). Resulting RNA products were separated on 6% polyacrylamide/7 M urea gels and eluted in TNES buffer (0.1 M Tris-HCl pH 7.5, 0.3 M NaCl, 0.01 M EDTA, 2% w/v SDS) overnight at room temperature. GST-Noc (final concentration 0.026 µg/µl) was incubated with purified [α-^32^P]-ATP-labeled RNA (final concentration 0.015 µM-0.18 µM) at 37°C for 30 min. 1 µl of the stopped reaction was spotted on a PEI cellulose plate, and developed in 15∶1∶10 isobutyric acid∶NH_4_OH∶H_2_O as the first dimension solvent, 0.75 M KH_2_PO_4_ as the second dimension solvent. Cold 5′AMP were analyzed on the same plate as a marker, and the position of it was determined under 254 nm UV light.

### Polysome analysis

Polysome analysis was conducted as described previously [45]. For the EDTA group, NIH3T3 cells were harvested in polysome extraction buffer (10 mM Tris-HCl, pH 7.4; 1% TritonX-100; 15 mM MgCl_2_; 0.3 M NaCl; 1 mg/mL Heparin) with 30 mM EDTA. For the cycloheximide group, cells were incubated in medium containing 0.1 mg/mL cycloheximide for 3 min before harvesting and were collected in 1 ml of polysome extraction buffer with 0.1 mg/mL cycloheximide. Sample was centrifuged at 12,000×g at 4°C for 10 min and the supernatant was loaded on a 10–50% consistency sucrose gradient and centrifuged at 35,000 rpms at 4°C for 190 min in an SW41 rotor. The gradient and fraction collection were done in a Gradient Station (BioComp). Tubulin and ribosomal P antigen were blotted as markers of non-polysome fractions and polysome fractions respectively.

### Immunocytochemistry

For Nocturnin subcellular localization, NIH3T3 cells were transfected with NOC-FLAG only (arsenite–induced P-bodies) or pE-eGFP-G3BP1 and NOC-FLAG (G3BP1–induced stress granules) constructs using Fugene6 transfection reagent (Roche). The α-FLAG antibody (Sigma) and α-GE-heds (PB marker; Santa Cruz) antibodies were used for staining. Immunodetection of stress granules and P-bodies was conducted according to Kedersha and Anderson [46].

### Statistics

For *Nocturnin* mRNA induction, one-way ANOVA was used to determine the induction. For *iNOS* mRNA two-way ANOVA followed by Bonferroni post-tests were used to determine the induction. For the mRNA half-life data, the Global Curve Comparison (Prism) was used. For iNOS protein induction and “sickness” score comparison, we used two-tailed student's T-test. For circadian profiles, rhythmicity of curves was determined by CircWave v1.4 software [http:/hutlab.nl; R. A. Hut, University of Groningen; [47]] For survival curve comparison, we used Log-rank (Mantel-Cox) test (Prism).
